# Rollout of dolutegravir-based antiretroviral therapy in sub-Saharan Africa and its public health implications

**DOI:** 10.11604/pamj.2020.37.243.25512

**Published:** 2020-11-17

**Authors:** Herieth Ismael Wilson, Herry Mapesi

**Affiliations:** 1 Ifakara Health Institute, Ifakara Branch, Ifakara, United Republic of Tanzania,; 2 Swiss Tropical and Public Health Institute, Basel, Switzerland,; 3 University of Basel, Basel, Switzerland

**Keywords:** HIV/AIDS, dolutegravir, pharmacovigilance

## To the editors of the Pan African Medical Journal

Globally, approximately 33 million people are living with Human Immunodeficiency Virus (HIV) and more than 60% of them live in sub-Saharan Africa. Widespread availability of antiretroviral treatment (ART) has reduced morbidity and mortality among people living with HIV (PLHIV). The increase in life expectancy in PLHIV has been associated with an increased burden of non-communicable diseases (NCDs), mainly being metabolic disorders [1]. PLHIV have an increased risk of developing NCDs since traditional risk factors for NCDs such as obesity, genetic predisposition and sedentary life intersect with HIV-specific risk factors such as long-term exposure to ART and chronic inflammation [2]. Integrase-strand-transfer inhibitors (INSTI)-based regimens have recently been rolled out as the new first-line treatment in most low and middle-income countries due to their excellent safety profile, lower price, and sustained treatment success compared to the currently used ART regimens [3]. In sub-Saharan Africa, most of the current guidelines recommends the use of dolutegravir-based regimens as the first line ART among PLHIV [3]. However, there is a lack of studies to evaluate long-term side effects of dolutegravir-based regimens among PLHIV living in low and middle-income countries.

**Dolutegravir-based regimens and excess weight gain:** recent studies have demonstrated excessive weight gain among PLHIV switched from other ART regimens such Non-Nucleoside Reverse Transcriptase Inhibitors (NNRTI)-based regimens to dolutegravir-based regimens [4]. The same effect has been observed among ART-naïve patients starting treatment with dolutegravir-based regimen compared to patients starting treatment with NNRTI-based regimens [5]. Although short-term weight gain is a positive prognostic factor for PLHIV, who are underweight due to advanced HIV disease, the development into obesity increases the risk of developing cardiovascular diseases [6]. As a consequence, the current World Health Organization guidelines cautioned clinicians about the potential implications of possible weight gain associated with dolutegravir-based regimens [7].

**Dolutegravir-based regimens and neural tube defects:** in 2018, there was a safety signal alert from Botswana Tsepamo birth-outcome surveillance study, which results indicated an increased absolute risk of neural-tube defects in infants born to women who used dolutegravir-based regimen at conception compared to those who used other non-dolutegravir-based regimen [8]. The public release of this report by drug monitoring authorities led to uncertainty about the use of dolutegravir-based regimen among women of reproductive potential. In The New England Journal of Medicine, Rebecca Zash and colleagues reported the follow-up results from the same surveillance demonstrating a potential association between dolutegravir exposure at conception and the development of neural-tube defects among women of childbearing potential [9].

What lessons do we take from the previous studies evaluating potential side effects of using dolutegravir-based regimens in PLHIV? First, it is currently unknown whether the increased risk of developing excess weight gain among ART-naïve patients starting a dolutegravir-based regimen will be observed also sub-Saharan African settings, specifically in rural environments, where the living conditions and comorbidities might be quite different. The risk factors for developing excessive weight gain include black ethnicity, being a woman, low CD4 count and high HIV viral load [4,5]. This is of particular concern since in sub-Saharan Africa, majority of PLHIV are women, and still patients present to the health care facilities with advanced HIV disease with low CD4 count, and high HIV viral load. Second, PLHIV with viral suppression from older ART regimen such as NNRTI have an increased risk of developing excess weight gain once switched to dolutegravir-based regimen [4]. Third, there is a potential risk of developing neural-tube defects among women who were using dolutegravir-based regimen during conception.

Despite the urgent need to introduce new ART in sub-Saharan Africa, establishment of proper pharmacovigilance systems is essential for monitoring the safety of these new medications. In order to detect rare events such as short-term weight gain and development of neural-tube defects, a large number of exposures is needed, which is possible only if after introduction of new drugs in the community and systematic reporting is done. Implementing improved pharmacovigilance systems will not only help to monitor the safety of new antiretroviral drugs, but also to monitor medications from other chronic diseases requiring lifelong treatment such as hypertension, diabetes mellitus, cancers and epilepsy, all of which are on the rise in low-income and middle-income countries. Furthermore, it will provide data on pharmacokinetics and safety in pregnancy since this information is usually available on average six years after registration of the new drug [10].

## Conclusion

It is vital that low-income and middle-income countries improve their pharmacovigilance systems to produce robust and high-quality evidence to monitor the safety of new drugs. Additionally, there is an urgent need for longitudinal post-licensing studies to evaluate potential mechanisms of newly detected adverse events such as the dolutegravir signal for a possible neural-tube defect, which might be easier to capture in settings with a high number of HIV-positive women in childbearing age.
